# Nonmuscle myosin heavy chain IIA facilitates SARS-CoV-2 infection in human pulmonary cells

**DOI:** 10.1073/pnas.2111011118

**Published:** 2021-12-06

**Authors:** Jian Chen, Jun Fan, Zhilu Chen, Miaomiao Zhang, Haoran Peng, Jian Liu, Longfei Ding, Mingbin Liu, Chen Zhao, Ping Zhao, Shuye Zhang, Xiaoyan Zhang, Jianqing Xu

**Affiliations:** ^a^Zhongshan Hospital, Institutes of Biomedical Sciences, Fudan University, Shanghai 201508, China;; ^b^Shanghai Public Health Clinical Center, Fudan University, Shanghai 201508, China;; ^c^Department of Microbiology, Second Military Medical University, Shanghai 200433, China

**Keywords:** SARS-CoV-2, MYH9, virus entry, COVID-19, pan-coronavirus

## Abstract

Severe acute respiratory syndrome coronavirus 2 (SARS-CoV-2), the causative agent of coronavirus disease 2019 (COVID-19), binds to host receptor angiotensin-converting enzyme 2 (ACE2) through its spike (S) glycoprotein, which mediates membrane fusion and viral entry. However, the expression of ACE2 is extremely low in a variety of human tissues, especially in the airways. Thus, other coreceptors and/or cofactors on the surface of host cells may contribute to SARS-CoV-2 infection. Here, we identified nonmuscle myosin heavy chain IIA (MYH9) as an important host factor for SARS-CoV-2 infection of human pulmonary cells by using APEX2 proximity-labeling techniques. Genetic ablation of MYH9 significantly reduced SARS-CoV-2 pseudovirus infection in wild type (WT) A549 and Calu-3 cells, and overexpression of MYH9 enhanced the pseudovirus infection in WT A549 and H1299 cells. MYH9 was colocalized with the SARS-CoV-2 S and directly interacted with SARS-CoV-2 S through the S2 subunit and S1-NTD (N-terminal domain) by its C-terminal domain (designated as PRA). Further experiments suggested that endosomal or myosin inhibitors effectively block the viral entry of SARS-CoV-2 into PRA-A549 cells, while transmembrane protease serine 2 (TMPRSS2) and cathepsin B and L (CatB/L) inhibitors do not, indicating that MYH9 promotes SARS-CoV-2 endocytosis and bypasses TMPRSS2 and CatB/L pathway. Finally, we demonstrated that loss of MYH9 reduces authentic SARS-CoV-2 infection in Calu-3, ACE2-A549, and ACE2-H1299 cells. Together, our results suggest that MYH9 is a candidate host factor for SARS-CoV-2, which mediates the virus entering host cells by endocytosis in an ACE2-dependent manner, and may serve as a potential target for future clinical intervention strategies.

## Significance

SARS-CoV-2 primarily targets the lung and enters the body through ACE2 receptors. However, the expression of ACE2 is extremely low in the airways. In this study, we utilized APEX2 proximity-labeling techniques and identified nonmuscle myosin heavy chain IIA (MYH9) as an ACE2 coreceptor to promote SARS-CoV-2 infection of human lung cells, as well as infection of pan-coronavirus. We demonstrated that MYH9 colocalized with SARS-CoV-2 S primarily at the membrane, which interacts with the S2 subunit and the S1-NTD subunit through its C-terminal domain, and enhances SARS-CoV-2 entry in an ACE2-dependent manner. Our results define MYH9 as a key host factor that facilitates SARS-CoV-2 and pan-coronavirus infection, which may serve as a potential target for future clinical intervention strategies.

Severe acute respiratory syndrome coronavirus 2 (SARS-CoV-2) has triggered a pandemic coronavirus disease (COVID-19) (1–3), resulting in more than 209 million infections worldwide since December 2019. SARS-CoV-2 is the seventh coronavirus (CoV) infecting humans beyond endemic human CoVs (HCoV-HKU1, -NL63, -OC43, and -229E), SARS-CoV, and Middle East respiratory syndrome CoV (MERS-CoV). SARS-CoV-2 preferentially infects the airway epithelial cells (1, 4), although viral infection has been detected in a variety of human organs, including the lungs, pharynx, heart, liver, brain, kidneys, and digestive system organs (5–7), causing upper respiratory diseases, fever, and severe pneumonia in humans.

**Fig. 1.**
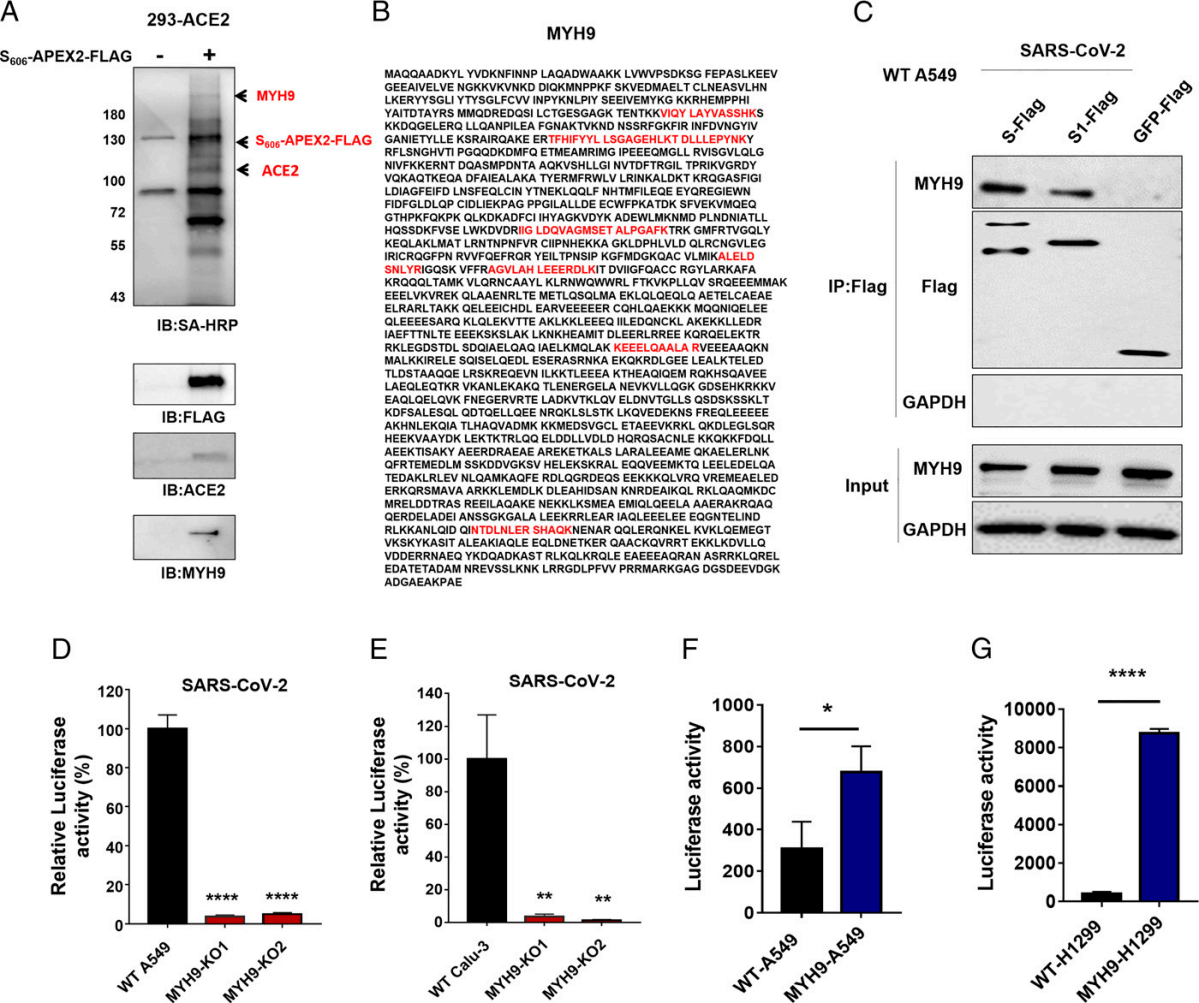
S606-APEX2-FLAG proximal labeling identifies MYH9 as a host factor for SARS-CoV-2 infection in human pulmonary cells. (*A*) Precipitation of the 293-ACE2 cell lysates without (line1) or with S606-APEX2-FLAG (lane 2) treatment. Beads were washed and eluted in the sample buffer, separated by SDS-PAGE, and analyzed by Western blotting using the HRP-conjugated streptavidin. Samples were also analyzed by Western blotting using specific antibodies to confirm the pull-down of S protein, ACE2, and MYH9. (*B*) The mass spectrometric analysis revealed the 226-kDa band MYH9 as a protein associated with the S protein. The peptides detected are highlighted (Red) in the protein sequence. (*C*) Immunoprecipitation with Flag-SARS-CoV-2 S, S1, and GFP as bait, followed by immunoblotting with anti-Flag, anti-MYH9, or anti-GAPDH antibodies. (*D–E*) Infection of SARS-CoV-2 pseudovirus in WT or MYH9-KO A549 (*D*) and Calu-3 cells (*E*). Significant differences from the WT cells were determined by two-tailed unpaired *t* test. ****P < 0.0001; **P < 0.01. (*F* and *G*) Luciferase activities in WT and MYH-A549 or MYH-H1299 cells 48 h post SARS-CoV-2 pseudovirus infection. Significant difference from WT cells were determined by two-tailed unpaired *t* test. ****P < 0.0001; *P < 0.05. All data in this figure are represented as means ± SEM of over three independent experiments.

CoVs were not considered to be highly pathogenic to humans until the outbreak of SARS-CoV started in 2002 in Guangdong province, China (8–10). The primary determinant of CoV tropism is their spike (S) glycoproteins, which mediate the viral infection by binding to membrane receptors on the host cells and could be cleaved to the N-terminal S1 and C-terminal S2 subunit by the host proteases such as transmembrane protease serine 2 (TMPRSS2) and Furin (11). Studies have shown that ACE2, a cellular receptor for SARS-CoV, also binds SARS-CoV-2 S and serves as the entry point for SARS-CoV-2 (3, 12). However, ACE2 cannot fully explain the tissue tropism of SARS-CoV-2 because of the virus detection in tissues with little ACE2 expression, such as the liver, brain, and blood, and even in the lung, only a small subset of cells expresses ACE2 (*SI Appendix*, Fig. 1*B*) (13–16). Recently, neuropilin-1 (NRP1) (17, 18) and heparan sulfate (19, 20) have been identified as cofactors implicated in enhancing ACE2-dependent SARS-CoV-2 infection, while tyrosine-protein kinase receptor (AXL) (21) and CD147 (22) were identified as receptors involved in SARS-CoV-2 cell entry independently of ACE2. These highlight the multiple routes of SARS-CoV-2 infection, likely contributing to high infectivity and spread of COVID-19. Hence, SARS-CoV-2 is assumed to be dependent on other potential receptors or coreceptors to facilitate its infection in humans.

Here, we applied engineered ascorbate peroxidase APEX2-based subcellular proteomics (23–25) to capture spatiotemporal membrane protein complexes interacting with SARS-CoV-2 S in living cells. We found that the nonmuscle myosin heavy chain IIA (MYH9) specifically interacts with SARS-CoV-2 S protein. The C-terminal domain (designated as PRA) of MYH9 interacts with the S2 subunit and the N-terminal domain (NTD) of the S1 subunit, and PRA overexpression facilitates a pan-CoV entry into host cells. In addition, depletion of MYH9 significantly reduced authentic SARS-CoV-2 infection in human lung cell lines. Mechanistically, a myosin inhibitor but not TMPRSS2 and CatB/L inhibitors blocked SARS-CoV-2 infection in PRA-A549 cells, and the presence of ACE2 is required for MYH9 mediated SARS-CoV-2 entry. Collectively, our results suggest that MYH9 is a coreceptor of ACE2 for SARS-CoV-2 infection in human pulmonary cells.

## Results

### APEX-Based Proteomics Reveal that MYH9 Is in Close Proximity to SARS-CoV-2 Spike Protein.

To identify the host proteins responsible for SARS-CoV-2 attachment and/or entry, we utilized an ascorbate peroxidase, APEX2-based, proximity-labeling technology to capture the local interactome of APEX2-tagged SARS-CoV-2 S protein (23, 24). The addition of biotin-phenol and hydrogen peroxide to cells induces the local biotinylation of proteins within a range of ∼20 nm of the APEX2-tagged protein. These biotinylated proteins were subsequently affinity purified via streptavidin beads and identified via mass spectrometry (MS). Gel electrophoresis followed by silver staining (*SI Appendix*, Fig. 1*A*) and anti-streptavidin/HRP Western blotting revealed many biotinylated proteins in ACE2-293T cell lysates treated with S606-APEX2-FLAG but not in controls without APEX2 (Fig. 1*A*). A protein of ∼220 kDa (Fig. 1*A*) was pulled down and identified by Liquid chromatography-tandem MS (LC-MS/MS) as nonmuscle MYH9 (Fig. 1*B* and *SI Appendix*, Table 1). To verify the association of SARS-CoV-2 S with MYH9, coimmunoprecipitation assays were conducted using A549 cells transfected with FLAG-tagged SARS-CoV-2 S or S1, with FLAG-tagged GFP as the negative control (NC). Immune precipitation with an anti-FLAG antibody identified an association of S or S1 with endogenous MYH9 in A549 cells (Fig. 1*C*). These data indicate that SARS-CoV-2 S may interact with endogenous MYH9 in human lung cells.

### MYH9 Facilitates SARS-CoV-2, SARS-CoV-1, and MERS-CoV Virus Entry into Human Pulmonary Cells.

**Fig. 2.**
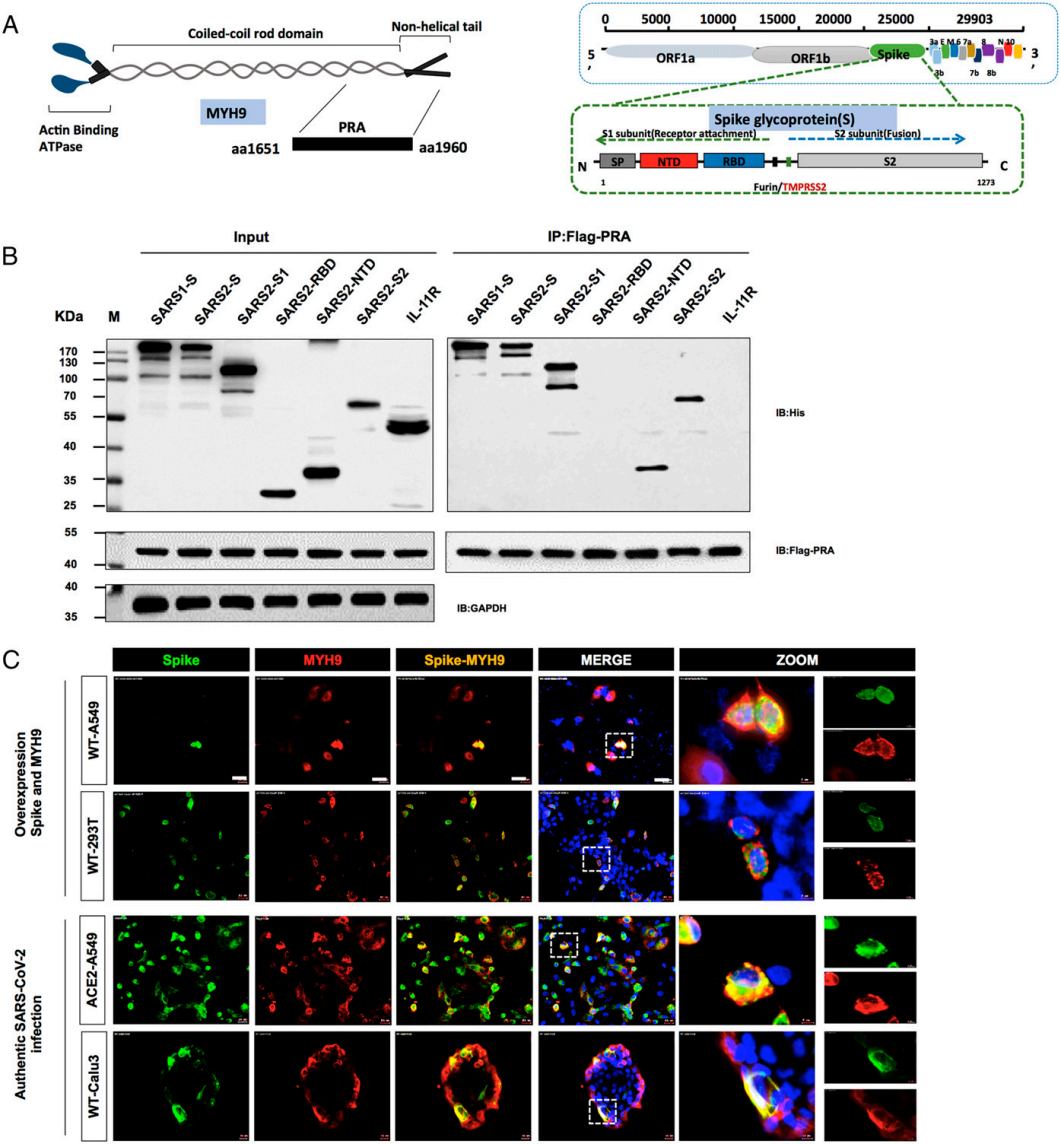
The SARS-CoV-2 S glycoprotein interacts with host MYH9. (*A*) Schematic illustration shows the PRA domain located within the full-length MYH9 protein and the SARS-CoV-2 spike protein. (*B*) The interaction between MYH9 and SARS-CoV-2 spike proteins. Flag-PRA 293T cells were transfected with His-tagged SARS-CoV-1 S, SARS-CoV-2 S, S1, S2, S1-RBD, S1-NTD, and IL-11R proteins as indicated, and IL-11R was an NC. Immunoprecipitation with Flag-PRA as a bait, followed by immunoblotting with anti-Flag, anti-His, or anti-GAPDH antibodies. All experiments were conducted more than three times. (*C*) Colocalization assay of SARS-CoV-2 S and MYH9. SARS-CoV-2 and MYH9 were cotransfected to WT A549 and WT 293T cells. ACE2-A549 and WT Calu-3 cells were infected with authentic SARS-CoV-2. Green, SPIKE; Red, MYH9; and Blue, DAPI. (Scale bars, 20 μm.)

MYH9 belongs to the myosin family, which has been identified as functional receptors for herpes simplex virus-1 (HSV-1) (26), Epstein–Barr virus (EBV) (27), and Porcine Reproductive and Respiratory Syndrome Virus (PRRSV) (28). MYH9 is highly expressed in most human tissues, particularly in human lungs (*SI Appendix*, Fig. 1*B*). To determine whether MYH9 is necessary for SARS-CoV-2 infection, we first knockout MYH9 in A549 and Calu-3 cells using CRISPR-Cas9 (Fig. 1*D*). The gene disruption efficiency was validated in A549 and Calu-3 cells (*SI Appendix*, Fig. 2 *A* and *B*). We found that the depletion of endogenous MYH9 not only significantly reduced the infection of SARS-CoV-2 pseudovirus (Fig. 1 *D* and *E*) but also reduced infection by the SARS-CoV-1 and MERS-CoV pseudovirus (*SI Appendix*, Fig. 2 *C* and *D*). Furthermore, exogenous overexpression of MYH9 significantly increased SARS-CoV-2 infection in A549 and NCI-H1299 cells (Fig. 1 *F* and *G*). Together, these results suggest that MYH9 plays an important role in SARS-CoV-2 infection of human pulmonary cells.

### The SARS-CoV-2 S Glycoprotein Interacts with Host MYH9.

**Fig. 3.**
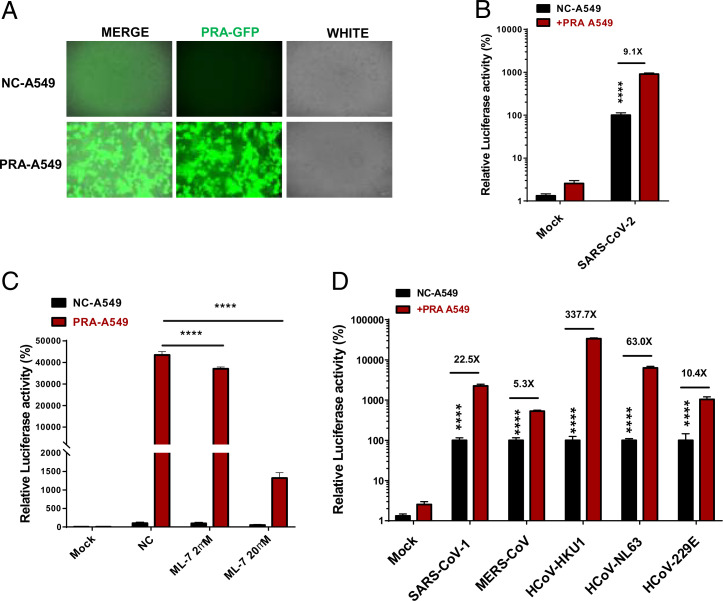
MYH9-PRA promotes pan-coronovirus pseudotype infection. (*A*) PRA expression levels were evaluated by GFP-tag expression using fluorescence microscopy. Green: PRA-GFP. (*B*) Relative SARS-CoV-2 pseudovirus infection in WT and PRA-A549 cells. Two-way ANOVA was performed with Tukey’s correction for multiple comparisons. ****P < 0.0001. (*C*) Pseudovirus infection of SARS-CoV-2 in PRA-A549 cells pretreated or untreated with different concentrations of myosin inhibitor ML-7. Two-way ANOVA was performed with Tukey’s correction for multiple comparisons. ****P < 0.0001. (*D*) Pseudoviral infections of pan-CoVs in NC- and PRA-A549 cells, including SARS-CoV-1, MERS-CoV, HCoV-HKU1, HCoV-NL63, or HCoV-229E. Significant differences from NC-A549 cells were determined by two-way ANOVA with Sidak’s multiple comparisons test. ****P < 0.0001. All data are represented as means ± SEM from over three independent experiments.

The amino-terminal head domain of MYH9 possesses ATPase activity and contains binding sites for actin and myosin lightchains (29, 30), while the MYH9 C-terminal domain (PRA) (Fig. 2*A*) interacts with viral glycoprotein 5 (GP5), a major protein in the PRRSV envelope, facilitating PRRSV infection (28). To determine whether the C-terminal domain is the key functional domain of MYH9 in SARS-CoV-2 infection, we first examined the interaction between PRA and SARS-CoV-2 S. Flag-tagged PRA-GFP was stably expressed in 293T cells, and a cell clone (#1) with strong PRA expression was isolated by Fluorescence Activating Cell Sorter (FACS) sorting. His-tagged SARS-CoV-1 S, or SARS-CoV-2 S, S1-full, S1-RBD (receptor-binding domain), S1-NTD, and S2-full were subsequently overexpressed in PRA-293T cells respectively. As a control, we cotransfected the Flag-tagged GFP and SARS-CoV-2 proteins into 293T cells (*SI Appendix*, Fig. 3). Notably, Flag-tagged PRA immunoprecipitated SARS-CoV-1 S, SARS-CoV-2 S, S1-subunit, S1-NTD, and S2-subunit but not S1-RBD (Fig. 2*B*), indicating that the PRA interacted with the S1-NTD and the S2 subunit rather than the S1-RBD. In addition, immunofluorescent staining revealed that MYH9 and SARS-CoV-2 S colocalized mainly to the cell membrane during transfection and authentic viral infection (Fig. 2*C*).

### PRA Facilitates Pan-CoV Infection.

To assess the role of PRA in SARS-CoV-2 infection, the pHAGE-CMV-2×Flag-PRA or the pHAGE-CMV-2×Flag-IRES-puro empty vector (NC) were overexpressed in the A549 cells prior to infection. Cells with high PRA levels were sorted by FACS (Fig. 3*A*). Infection with SARS-CoV-2 pseudovirus was significantly increased in PRA-expressing A549 cells (Fig. 3*B*). Furthermore, the pseudoviral infection was drastically inhibited by myosin inhibitor ML-7 in PRA-expressing A549 cells (Fig. 3*C*), suggesting the implication of the C-terminal domain of MYH9 in promoting SARS-CoV-2 infection in human lung epithelial cells. In addition to SARS-CoV-2 and SARS-CoV-1, other CoVs such as α-CoVs (HCoV-229E and HCoV-NL63) and β-CoVs (HCoV-OC43, HCoV-HKU1, and MERS-CoV) also infect humans (31). Evolutionarily, the RBD of CoVs is part of highly mutable region. In contrast, the S2 subunit is relatively conserved among different CoVs and plays a pivotal role in CoVs’ infection by promoting viral fusion. The above data confirmed that MYH9-PRA can interact with the SARS-CoV-2 S2 subunit and S1-NTD domain (Fig. 2*B*). Thus, to test whether MYH9-PRA may promote infection of other CoVs in human lung cells, we infected NC and PRA-A549 cells with two αCoVs (HCoV-229E and HCoV-NL63) and three βCoVs (SARS-CoV-1, MERS-CoV, and HCoV-HKU1). We found that overexpression of PRA significantly enhanced pan-CoV infection in A549 cells (Fig. 3*D*). These results suggest that MYH9 may be a universal target for the prevention of CoVs infection.

### MYH9 PRA Promotes SARS-CoV-2 Entry in an ACE2-Dependent Manner and Bypasses TMPRSS2 and CatB/L Pathway.

**Fig. 4.**
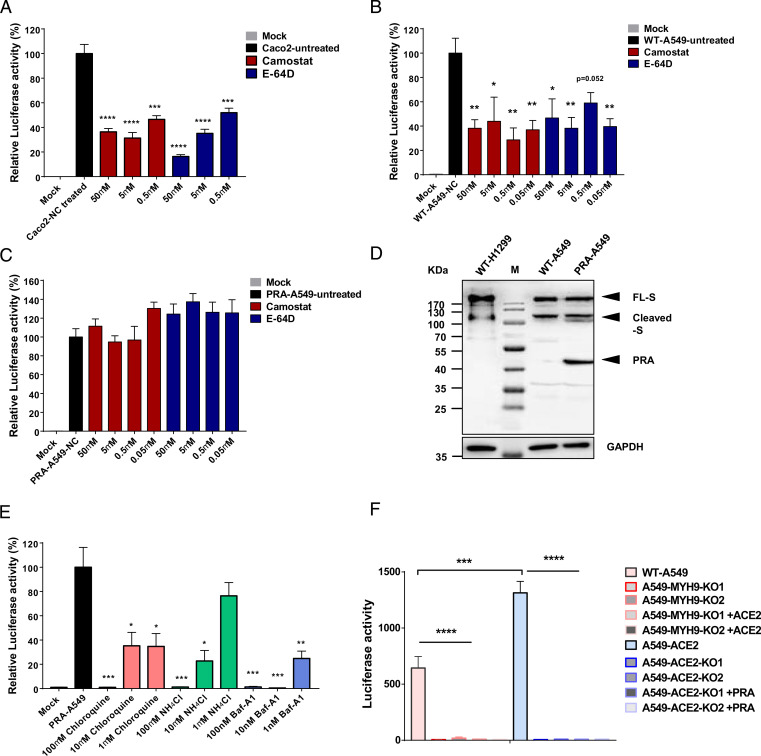
MYH9 PRA promotes SARS-CoV-2 pseudovirus entry and fusion independently from TMPRSS2 and CatB/L. (*A–C*) Pseudovirus infection of SARS-CoV-2 in Caco-2 (*A*), WT A549 (*B*), and PRA-A549 cells (*C*) pretreated with various concentration of camostat or E-64D to inhibit serine proteases or cathepsins, respectively. Significant differences from mock-treated cells were determined by two-tailed unpaired *t* test; ****P < 0.0001; ***P < 0.001; **P < 0.01; and *P < 0.5. (*D*) Cleavage of full length of SARS-CoV-2 S in WT H1299, WT A549, and PRA-A549 cells. (*E*) Relative SARS-CoV-2 pseudovirus infection of PRA-A549 cells pretreated with various concentration of Choroquine, NH_4_Cl, or Baf-A1 to inhibit endosome maturation. Significant differences from mock-treated cells were determined by two-tailed unpaired *t* test. ***P < 0.001; **P < 0.01; and *P < 0.5. (*F*) Luciferase activities in A549 cells 48 h post SARS-CoV-2 pseudovirus infection. Significant difference from WT or ACE2-A549 cells were determined by two-tailed unpaired *t* test. ****P < 0.0001; ***P < 0.001. All data in this figure are represented as means ± SEM from three independent experiments (*n* ≥ 4).

We next investigated protease dependence of SARS-CoV-2 entry in Caco-2 colon epithelial cells, wild type (WT) A549 cells and PRA-A549 pulmonary epithelial cells. CoVs enter cells through two pathways: plasma membrane fusion (early route) or endosome fusion (late route) in a cell type–dependent manner (32). The presence of exogenous and membrane proteases, such as trypsin and TMPRSS2, triggers the early fusion pathway. Otherwise, SARS-CoV-2 is endocytosed through the late pathway, cleaved by cathepsin B and L (CatB/L). To determine whether the PRA facilitated the entry of SARS-CoV-2 entry by TMPRSS2 or CatB/L pathway, we initially used camostat mesylate (a TMPRSS2 inhibitor) and E-64d (a CatB/L inhibitor) to block TMPRSS2 and CatB/L activity. We found that camostat mesylate and E-64d strongly inhibit SARS-CoV-2 (Fig. 4*A*) and SARS-CoV-1 pseudovirus infection (*SI Appendix*, Fig. 4) in Caco-2 cells and reduced SARS-CoV-2 infection in WT A549 cells (Fig. 4*B*). In contrast, the two inhibtors did not affect SARS-CoV-2 pseudovirus infection in PRA-A549 cells (Fig. 4*C*). We then expressed SARS-CoV-2 S in WT H1299, WT A549, and PRA-A549 cells separately and found that SARS-CoV-2 S was successfully cleaved in these cells (Fig. 4*D*), indicating that PRA antagonized the function of protease inhibitors and promoted the entry of SARS-CoV-2 through an alternative route. To determine whether the endocytic pathways were involved in MYH9-PRA–mediated SARS-CoV-2 entry, we then tested the agents affecting endocytosis over SARS-CoV-2 infection. Inhibition of endosome maturation with a range of concentrations of ammonium chloride (NH_4_Cl), Bafilomycin A1 (Baf-A1), or Chloroquine (Chloq) greatly reduced SARS-CoV-2 pseudovirus infection (Fig. 4*E*). These data suggest that MYH9-PRA promotes SARS-CoV-2 entry and fusion but bypasses TMPRSS2 and CatB/L pathway.

**Fig. 5.**
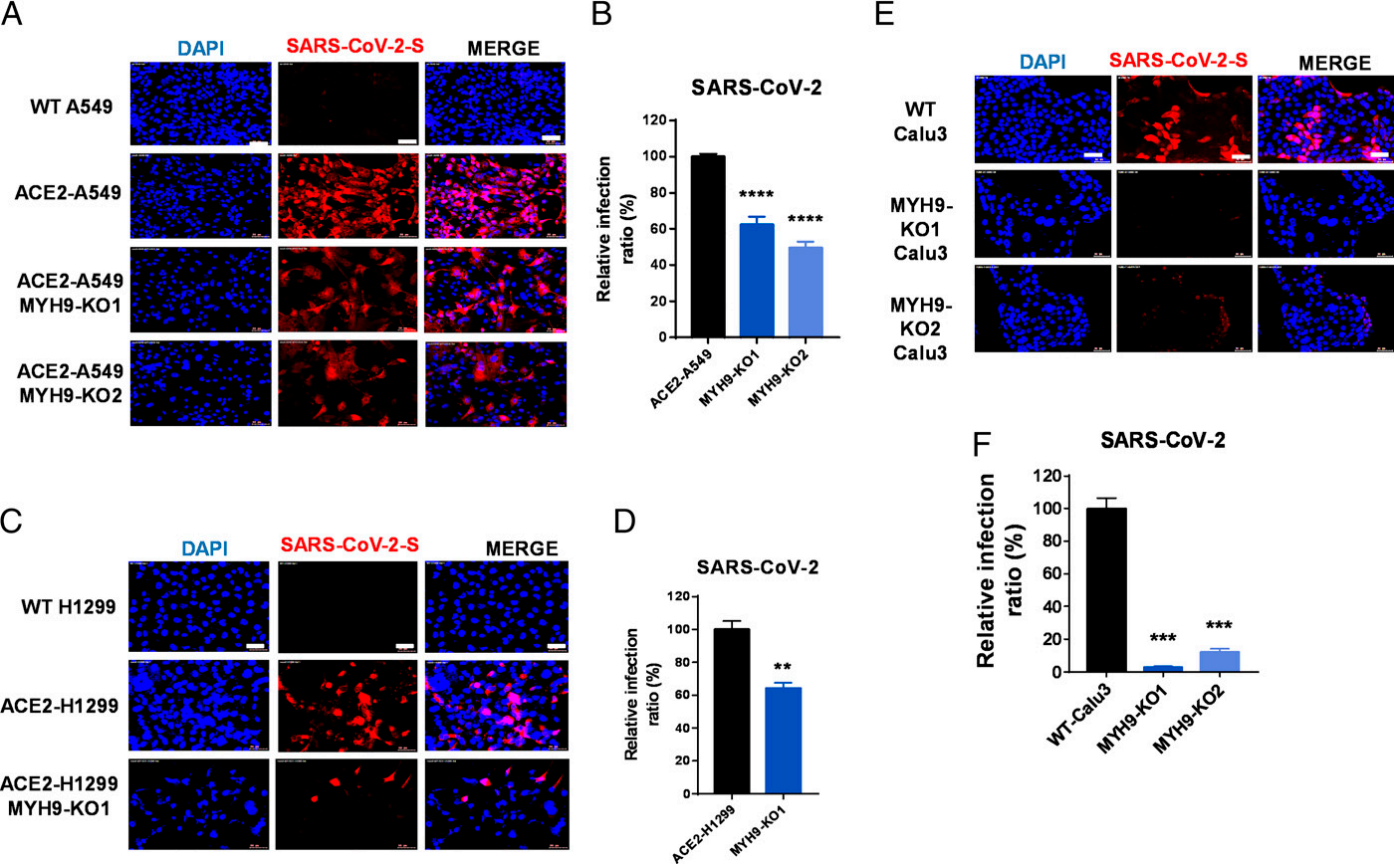
MYH9 promotes authentic SARS-CoV-2 infection in human pulmonary epithelial cells. (*A–B*) Authentic SARS-CoV-2 infection in WT A549, ACE2-expressing A549, or MYH9-KO ACE2-A549 cells. (*A*) Immunofluorescence images representative of three independent experiments. (Scale bars, 50 μm.) (*B*) Statistical analyses. Each biological replicate (n = 3) contains 3,000 cells analyzed. ****P < 0.0001. Significantly different from WT cells (two-tailed Student’s *t* test). (*C* and *D*) Authentic SARS-CoV-2 infection in WT H1299, ACE2-expressing H1299, or MYH9-KO ACE2-H1299 cells. (*C*) Immunofluorescence images representative of three independent experiments. (Scale bars, 50 μm.) (*D*) Statistical analyses. Each biological replicate (n = 3) contains 3,000 cells analyzed. **P  < 0.01. Significant differences from WT cells were determined by two-tailed Student’s *t* test. (*E* and *F*) Authentic SARS-CoV-2 infection in WT Calu-3 cells and MYH9-KO Calu-3 cells. (*E*) Immunofluorescence images representative of three independent experiments. (Scale bars, 50 μm.) (*F*) Statistical analyses. Each biological replicate (n = 3) contains 3,000 cells analyzed. ***P < 0.001. Significant differences from WT cells were determined by two-tailed Student’s *t* test. The quantitative data in this figure are shown as the mean ± SEM. DAPI, blue; SARS-CoV-2 S, red.

To evaluate the importance of MYH9 in SARS-CoV-2 infection in pulmonary epithelial cells, we depleted ACE2 in PRA-A549 cells and found that the infection with SARS-CoV-2 was significantly decreased, suggesting that MYH9 might not act as a virus receptor alone and that low ACE2 expression is required for MYH9-mediated SARS-CoV-2 infection (Fig. 4*F*). Overexpression of ACE2 enhanced SARS-CoV-2 infection in A549 cells, while depletion of MYH9 in ACE2-A549 cells significantly reduced SARS-CoV-2 pseudovirus infection (Fig. 4*F*) or authentic SARS-CoV-2 infection (Fig. 5 *A* and *B*), indicating the important role of MYH9 acting as an ACE2 coreceptor during SARS-CoV-2 infection. Collectively, these results indicate that MYH9-PRA enhanced endocytic entry of SARS-CoV-2 in ACE2-dependent manner but is independent of TMPRSS2 and CatB/L.

### MYH9 Promotes Authentic SARS-CoV-2 Infection in Human Pulmonary Cells.

To determine whether MYH9 promotes authentic SARS-CoV-2 virus infection, we first infected wild-type A549 and H1299 cells with authentic SARS-CoV-2. Consistent with previous study (33), we found that neither of these cells have been successfully infected 48 h post inoculation (Fig. 5 *A* and *C*). Hence, we generated A549 and H1299 cells overexpressing ACE2-GFP, by sorting the GFP positive cells three times using FACS. The overexpression of ACE2 greatly enhanced authentic SARS-CoV-2 virus infection in A549 and H1299 cells (Fig. 5 *A* and *C*). Depletion of MYH9 in the ACE2-A549 and ACE2-H1299 cells significantly reduced authentic SARS-CoV-2 virus infection (Fig. 5 *B* and *D*), indicating that MYH9 is needed for SARS-CoV-2 infection in ACE2-expressed human lung cells. In addition, we studied the role of MYH9 in Calu-3 cells, a pulmonary cell line compatible with SARS-CoV-2 infection. Knocking out MYH9 in the Calu-3 cells greatly reduced authentic SARS-CoV-2 virus infection (Fig. 5 *E* and *F*). Together, these data indicate that MYH9 is required for SARS-CoV-2 infection in human pulmonary cells.

## Discussion

The COVID-19 pandemic has infected 209 million people and is now a global public health crisis, causing severe respiratory tract infections in humans. Treatment options for COVID-19 are still limited. Although vaccines to prevent SARS-CoV-2 infection are currently partially available across most countries and regions, SARS-CoV-2 is still widespread with significant morbidity and mortality. ACE2 has been widely acknowledged as a receptor for CoV infection, including SARS-CoV-2 (3, 12, 34). However, depletion of AXL but not ACE2 reduced SARS-CoV-2 infection in human pulmonary cells (21). Actually, AXL may promote Zika virus infection by antagonizing interferon (IFN) signaling in human astrocytes (35). Consistent with our findings, recent studies revealed that RIG-I-KO A549 cells were more susceptible to SARS-CoV-2 infection than the wild-type A549 cells (33). Hence, it remains to be determined in further studies whether AXL and ACE2 promote SARS-CoV-2 replication by down-regulation of IFN signaling. In addition, the entry of SARS-CoV-2 into cells markedly down-regulates the expression of ACE2, thereby promoting the progression of inflammatory and thrombotic processes. Therefore, it is unlikely that the treatment by blocking the ACE2 receptor will work (36). In our study, we discovered that MYH9, highly expressed in human lungs, is a host factor for SARS-CoV-2 infection and could be a target for COVID-19 prevention. Our data showed that MYH9 promotes SARS-CoV-2 infection in human lung cell lines, such as Calu-3, A549 cells, and H1299 cells and functions as an ACE2 cofactor for the entrance of SARS-CoV-2. Indeed, we found that MYH9 does not enhance SARS-CoV-2 pseudovirus infection in ACE2-depleted A549 cells but rather increases viral infection in WT A549 cells. Importantly, genetic ablation of MYH9 significant reduced SARS-CoV-2 pseudovirus and authentic SARS-CoV-2 infection in ACE2-A549 cells, indicating that MYH9 does not act alone as a virus receptor but acts as a coreceptor for ACE2 when ACE2 expression is low. Nevertheless, MYH9 may be considered as another candidate therapeutic target for SARS-CoV-2, and further studies are needed to clarify the underlying mechanisms of cooperation between ACE2 and MYH9 during SARS-CoV-2 infection.

Myosins are a large family of motor proteins that share the common features of ATP hydrolysis (ATPase enzymatic activity), actin binding, and kinetic energy transduction potential. Myosin-10 is an important paralogue of MYH9 and is involved in viral uncoating when the Influenza A virus (IAV) entered through endocytosis (37). Similarly, MYH9 promotes viral entry and infection by interacting with gB of HSV-1, Gn of SFTSV, and gH/gL of EBV in permissive cells (26–28). Our results demonstrated that MYH9 colocalized with SARS-CoV-2 S primarily to the membrane. These data indicate that myosins may mediate IAV and other viruses infecting host cells during the entry steps. Coronaviruses enter cells through two pathways: plasma membrane fusion (triggered by FURIN or TMPRSS2) or endosome fusion (triggered by CatB/L) depending on the cell type (32, 38, 39). In the current study, we found that full length of SARS-CoV-2 S was successfully cleaved in WT or PRA-A549 cells. Inhibition of TMPRSS2 and CatB/L reduced SARS-CoV-2 entry in WT A549 cells but did not affect the entry of SARS-CoV-2 in MYH9-PRA A549 cells, whereas the endosomal pathway inhibitors, including NH_4_Cl, Baf-A1, and Chloq, significantly reduced the entry of SARS-CoV-2, supporting a critical role for MYH9 in viral endosome entry and independently from TMPRSS2 or CatB/L. Collectively, MYH9 may facilitate the entry of SARS-CoV-2 by a new endosomal pathway, which depends on the ACE2 receptor, but not on TMPRSS2 and CatB/L. The TMPRSS2 inhibitor is a clinically approved drug and can block the entry of SARS-CoV-2, as a treatment option (12). Our current data shows that myosin inhibitor could reduce MYH9-mediated SARS-CoV-2 entry into cells, providing a therapeutic target for treating COVID-19.

It has been reported that the RBD of the S of SARS-CoV-1 and SARS-CoV-2 is responsible for binding to ACE2 receptor (40–43). Therefore, the effectiveness of human neutralizing antibodies elicited by SARS-CoV-2 infection is well correlated with their competition with ACE2 for RBD binding (44–47). Interestingly, NRP1, known to bind substrates cleaved by furin, significantly potentiates the infectivity of SARS-CoV-2, and the binding of NRP1 to the CendR peptide in subunit S1 is likely responsible for increased infectivity of SARS-CoV-2 compared to SARS-CoV (17, 18). Other recent studies identified potent neutralizing human antibodies that bind to the NTD of S1 subunit but not RBD (48). Moreover, another recent study showed that the human tyrosine-protein kinase receptor AXL promotes SARS-CoV-2 infection by binding the S1-NTD and enhances viral entry (21). These studies call for a more systematic study of how the different regions of the S protein and MYH9 interact. Here, we revealed that the C-terminal domain of MYH9 is likely to bind the NTD of SARS-CoV-2 S1 but not the RBD (48, 49) highlighted the important role of NTD in SARS-CoV-2 infection. In addition, the S protein of SARS-CoV-2 and SARS-CoV-1 may interact with the C-terminal domain of MYH9. These results provide clues about how MYH9 interact with pan-CoV spike proteins. Genome-wide CRISPR screening has identified and validated TMEM41B as a pan-CoV host factor required for a postentry stage in the coronavirus (50). In this study, we showed that MYH9 is a critical host factor for α-CoVs and β-CoVs and significantly facilitates viral entry into human lung cells, therefore making it an attractive target for further investigation.

Together, our findings demonstrate the essential role of MYH9 in facilitating SARS-CoV-2 infection. In addition, our data indicate that MYH9 may promote pan-CoV infection, further validation and mechanistic elucidation can guide the design of universal entry inhibitors for the control of pan-CoV infection.

## Materials and Methods

### Cell Lines and Viruses.

A549, NCI-H1299, 293T, and Caco2 cell lines were purchased from the American Type Culture Collection (ATCC). Calu-3 cell line was gifted from Zhengli Shi (Wuhan Institute of Virology, Wuhan, People's Republic of China). A549, Caco2, and 293T cells were cultured in Dulbecco's modified Eagle medium (DMEM) (Gibco) supplemented with 10% fetal bovine serum (FBS), 100 IU/mL of penicillin, and 100 µg/mL of streptomycin. NCI-H1299 cells were cultured in Roswell Park Memorial Institute (RPMI) 1640 (Gibco) supplemented with 10% FBS (Gibco), 100 IU/mL of penicillin, and 100 µg/mL of streptomycin. Calu-3 cells were grown and propagated in Dulbecco's modified Eagle's medium–nutrient mixture F-12 medium (Gibco) supplemented with 15% fetal bovine serum. All cells were maintained at 37 °C in a fully humidified atmosphere containing 5% CO_2_ and were tested by Saily Bio (Shanghai, China) and are free of mycoplasma contamination. SARS-CoV-2/SH01/human/2020/CHN (GenBank accession no. MT121215) was propagated and titrated in Vero E6 cells and used for authentic virus-based infection assay.

### Plasmids and Molecular Cloning.

SARS-CoV-2 S (C-His tag, VG40589-CH), S1 (C-His tag, VG40591-CH), S1-RBD (N-His tag, VG40592-NH), and S2 (C-His tag, VG40590-CH) Gene ORF (open reading frame) cDNA (complementary DNA) clone expression plasmid were purchased from Sino Biological. SARS-CoV-2 S1-NTD (C-His tag) and S1 (C-Flag tag) plasmids were synthesized directly. Human SARS coronavirus (SARS-CoV-1) S Gene ORF cDNA clone expression plasmid (C-His tag, VG40150-CH), MERS-CoV S Gene ORF cDNA clone expression plasmid (C-His tag, VG40069-CF), Human coronavirus (HCoV-229E) S Gene ORF cDNA clone expression plasmid (C-Flag tag, VG40605-CF), Human coronavirus (HCoV-NL63) S Gene ORF cDNA clone expression plasmid (C-Flag tag, VG40604-CF), Human coronavirus (HKU1) S Gene ORF cDNA clone expression plasmid (C-Flag tag, VG40021-NF), SARS-CoV-2 (2019-nCoV) S Gene ORF cDNA clone expression plasmid (C-Flag tag, VG40589-CF), Human IL11RA/IL-11RA transcript variant 1 Gene ORF cDNA clone expression plasmid (C-His tag, HG10252-CH), and ACE2 lentiviral cDNA ORF Clone (C-GFPSpark tag, Human, HG10108-ACGLN) mammalian expression plasmids were purchased from Sino Biological. pcDNA3.1+/C-(K)DYK-MYH9 mammalian expression plasmids were purchased from GenScript (NM_002473.6). To generate the C-terminal 2×Flag-tagged MYH9 lentiviral construct (pHAGE-MYH9-2×Flag), pHAGE-CMV-2×Flag-IRES-GFP was digested with NotI and XhoI. pHAGE-CMV-2×Flag-IRES was used as NC. Then full-length MYH9 was amplified from pcDNA3.1(+)-MYH9-DYK. These two fragments were homologously recombined with a ClonExpress II One Step Cloning Kit (Vazyme Biotech, C112-02) according to the manufacturer’s instructions to generate the final pHAGE-MYH9-2×Flag construct. Similarly, the PRA-tagged lentiviral construct was generated by replacing MYH9 with the complete PRA ORF (PCR from MYH9 plasmid) to generate pHAGE-PRA-2×Flag. Single guide RNAs (sgRNAs) against MYH9 (sg1-MYH9: 5′-ACGCCACGTACGCCAGATAC-3″, sg2-MYH9:5′-CACGTGCCTCAACGAAGCCT-3″) were synthesized and cloned into the pLenti-V2 vector. The lentivirus was packaged in human embryonic kidney 293T (HEK293T) cells, condensed by ultra-centrifugation, and used to infect WT A549, WT H1299, ACE2-A549, and ACE2-H1299 cells. The cells were selected with puromycin (2 μg/mL) for 14 d and subcloned to form single colonies. KO cells were validated by Western blotting assay to verify the loss of MYH9 expression.

### APEX2.

A total of 8 ×10^6^ 293T-ACE2 cells were harvested, washed with phosphate-buffered saline (PBS) for three times, and subsequently treated with or without S606-APEX2-FLAG for 2h at 4 °C. Freshly prepared biotin-phenol was added to PBS to yield a final concentration of 500 μM. After removal of treated proteins, cells were supplied with PBS-biotin-phenol for 5 min at 37 °C. Biotinylation was achieved by adding freshly prepared H_2_O_2_ at a final concentration of 1 mM for exactly 1 min. Reactions were halted by removing the media, transferring the cells on ice, and performing three washes, each wash for 5 min, in freshly prepared quencher buffer (1× PBS containing 10 mM sodium azide, 10 mM sodium ascorbate, and 5 mM Trolox). Then the cells were collected in the quencher buffer and spun down at 4 °C, and the cell pellets were further lysed in radio immunoprecipitation assay (RIPA) buffer containing quencher buffer. Supernatants from the cell lysates were then incubated with Streptavidin Meagnetic beads for 2 h at 4 °C on a rotating wheel. Beads were washed and eluted in sample buffer, separated by sodium dodecyl sulfate-polyacrylamide gel electrophoresis (SDS-PAGE), and analyzed by Western blotting using HRP-conjugated streptavidin. Samples were also analyzed by Western blotting using specific antibodies to confirm the pull-down of the proteins. The 226-kDa band enriched in S606-APEX2-FLAG–treated 293-ACE2 cells were analyzed using LC-MS/MS. Peptides detected are highlighted (Red) in the protein sequence.

### SARS-CoV-2 Virus Pseudotype Production and Infection.

SARS-CoV-2 virus pseudotype was produced by cotransfection 293T cells with pNL4-3-Luc-R-E- (a HIV-1 NL4-3 luciferase reporter vector that contains defective Nef, Env, and Vpr; the luciferase gene was inserted in the nef gene; Catalog no. 3418, NIH AIDS Reagent Program) and pCMV-SARS-CoV-2-S, SARS-CoV-S, MERS-CoV-S, HCoV-NL63-S, HCoV-229E-S, HCoV-HKU1-S, or pCMV-Myc as control by using TurboFect Transfection Reagent (Thermo Fisher Scientific). The supernatants were harvested at 48 h (h) posttransfection, passed through 0.45-μm filter, and centrifuged at 800 × g for 5 min to remove cell debris. To transduce cells with pseudovirions, Calu-3, A549, Caco2, or H1299 cells were seeded into 96-well plates and inoculated with 100 μL media containing pseudovirions. After 2 h incubation, cells were fed with fresh media. About 48 h post inoculation, cells were lysed with 50 μL medium containing 50% Steady-glo (promega) at room temperature for 10 min. The transduction efficiency was measured by quantification of the luciferase activity using a Modulus II microplate reader (Turner Biosystems, Sunnyvale). All experiments were done in more than triplicates and repeated at least twice or more.

### Immunoprecipitation Assay.

For coimmunoprecipitation experiments, we used two approaches to analyze the association of MYH9 and S. In the first approach, WT A549 cells (4 × 10^6^ cells per 10-cm dish) were transiently transfected with 14 µg of pCMV-SARS2-S-Flag, pCMV-SARS2-S1- Flag, or pCMV-GFP-Flag separately using TurboFect Transfection Reagent (Thermo Fisher Scientific). In the second approach, 293T-MYH9 PRA-Flag cells (1 × 10^7^ cells per 10-cm dish) were transiently transfected with 14 µg of pCMV-SARS1-S-His, pCMV-SARS2-S-His, pCMV-SARS2-S1-His, pCMV-SARS2-S-RBD-His, pCMV-SARS2-S-NTD-His, or pCMV-SARS2-S2-His separately using TurboFect Transfection Reagent (Thermo Fisher Scientific); WT 293T cells were cotransfected with pCMV-GFP-Flag and pCMV-SARS2 proteins above. Cells were rinsed twice with cold PBS and were then transferred to clean tubes and lysed in cell buffer for Western blotting and immunoprecipitation supplemented with 1% protease inhibitor mixture (catalog no. P8340, Sigma-Aldrich). Cell lysates were incubated with Pierce Protein A/G Agarose (Sigma-Aldrich, 20422) for 4 h at 4 °C and were then subjected to centrifugation at 10,000 × g for 10 min at 4 °C. The supernatant was transferred to a new tube and incubated with 30 µL anti-Flag M2 affinity gel (Sigma-Aldrich, A2220) overnight at 4 °C. The Sepharose samples were centrifuged and washed five times with cell lysis buffer and eluted using 3×Flag peptide (Sigma-Aldrich, F4799). Then all samples were boiled with SDS loading buffer for 10 min.

### Immunofluorescence Staining.

Cells on slides were fixed with 4% paraformaldehyde for 20 min at room temperature and were permeabilized with 0.1% Triton-X 100 in PBS for 5 min and blocked with blocking buffer (1% bovine serum albumin and 2% donkey serum diluted in PBS) for 30 min. Immunofluorescence analyses of SARS-CoV-2–infected cells were performed using a rabbit anti–SARS-CoV-2 spike S/S2 protein antibody (1:500, 40590-T62, S&B), a mouse anti-MYH9 protein antibody (10ug/mL, ab55456, Abcam), and Alexa Fluor 680 donkey anti-rabbit IgG (H+L) (1:1,000, ab175772, Abcam). All cells were mounted with ProLongTM Gold Antifade with DAPI (Life Technologies, P36931) and imaged with a TissueFAXS 200 flow-type tissue cytometer (TissueGnostics GmbH, Vienna, Austria). All statistical analyses of immunofluorescence staining present the results from at least 3,000 cells per replicate, and data are shown as the mean ± SEM.

### Western Blot Analysis.

Cells were lysed using 4×SDS loading buffer and denatured at 95 °C for 10 min. Protein samples were resolved by SDS-PAGE, transferred to polyvinylidene fluoride membranes (GE Healthcare), and processed for Western blotting. Western blot detection of MYH9 and ACE2 was performed using a rabbit anti-MYH9 antibody (1:1,000, A0173, Abclonal) and an anti-ACE2 antibody (1:1,000 dilution, A12737, Abclonal), with a goat anti-rabbit IgG-HRP antibody (1:3,000, B2615, Santa Cruz Biotechnology) as the secondary antibody. GAPDH was used as a loading control.

Other antibodies used in the study included: mouse anti-Flag M2 (1:2,000, F1804, Sigma-Aldrich), mouse anti His-tagged mAb (1:1,000, AE003, Abclonal), anti-GAPDH (1:3,000, AC002, Abclonal), goat anti-rabbit IgG-HRP (1:5,000, B2615, Santa Cruz Biotechnology), and goat anti-rabbit IgG-HRP (1:5,000, 31430, Invitrogen) antibodies.

### Authentic SARS-CoV-2 Infection of Human Cells.

Authentic SARS-CoV-2 was isolated from a patient in Shanghai with COVID-19. We plaque-purified and massively expanded the initial generation in Vero-E6 in the presence of *N*-tosyl-l-phenylalanine chloromethyl ketone-treated trypsin at concentration of 2 μg/mL and stored the virus at −80 °C. We deep-sequenced the strain and named it SARS-CoV-2/human/CHN/Shanghai_CH-03/2020 (GenBank accession no. MT622319.1). Compared with the Wuhan strain (GenBank accession no. NC_045512), this strain has the same gene sequence encoding the S glycoprotein. All authentic SARS-CoV-2 infection assays were performed using these early passages of SARS-CoV-2 to ensure the consistency of our experiments. WT or ACE2-expressed A549 or H1299 cells and Calu-3 cells were seeded in 8-well plates (4 × 10^4^ cells per well) in DMEM or DMEM/F12 containing 2% FBS. The cells were infected with 8 × 10^4^ pfu/mL SARS-CoV-2 (multiplicity of infection = 2) at 37 °C for 1 h, washed with 1× PBS three times, and cultured in complete medium for 48 h. All the experiments were performed in biosafety level 3 laboratories.

### Statistical Analyses.

All the Western blotting, luciferase assay, and immunofluorescence data were obtained from at least three repeated experiments. For quantification of infected cells, at least 1,000 fluorescent cells were imaged and counted with a flow-type tissue cytometer. More than three replicates were established per sample. The data were analyzed using Prism 7.0 software (GraphPad) and are presented as the means ± SEM. Statistical significance between two groups was determined by unpaired two-tailed Student’s *t* test. Multiple group comparisons were performed using two-way ANOVA. Differences were considered to be significant for *P* < 0.05 (indicated with an asterisk [*]).

## Supplementary Material

Supplementary File

## Data Availability

All data supporting the findings of this study are available within the article and *SI Appendix*.
