# Supplementary material for: Nonmuscle myosin heavy chain IIA facilitates SARS-CoV-2 infection in human pulmonary cells
Source: Proc Natl Acad Sci U S A. 2021 Dec 6;118(50):e2111011118. doi: 10.1073/pnas.2111011118 (PMC8685683; doi:10.1073/pnas.2111011118)
Supplement: Supplementary File [file pnas.2111011118.sapp.pdf]

Nonmuscle myosin heavy chain IIA facilitate SARS-CoV-2 infection in human pulmonary cells

Jian Chen[a,b,1], Jun Fan[b,1], Zhilu Chen[a,1], Miaomiao Zhang[b,1], Haoran Peng[c,1], Jian Liu[b], Longfei Ding[b], Mingbin Liu[b], Chen Zhao[b], Ping Zhao[c,2], Shuye Zhang[a,b,2], Xiaoyan Zhang[a,b,2], Jianqing Xu[a,b,2]

[a]Zhongshan Hospital, Institutes of Biomedical Sciences, Fudan University, Shanghai, 201508, China

[b]Shanghai Public Health Clinical Center, Fudan University, Shanghai, 201508, China

[c] Department of microbiology, Second military medical university, Shanghai 200433, China.

[1]Co-first author

[2]To whom correspondence may be addressed. **Email:** Ping Zhao（pnzhao@163.com (P.Z.), Shuye Zhang (zhangshuye@shphc.org.cn), Xiaoyan Zhang (zhangxiaoyan@shphc.org.cn),& Jianqing Xu (xujianqing@shphc.org.cn)）

This PDF file includes:

Figures S1 to S4

Tables S1

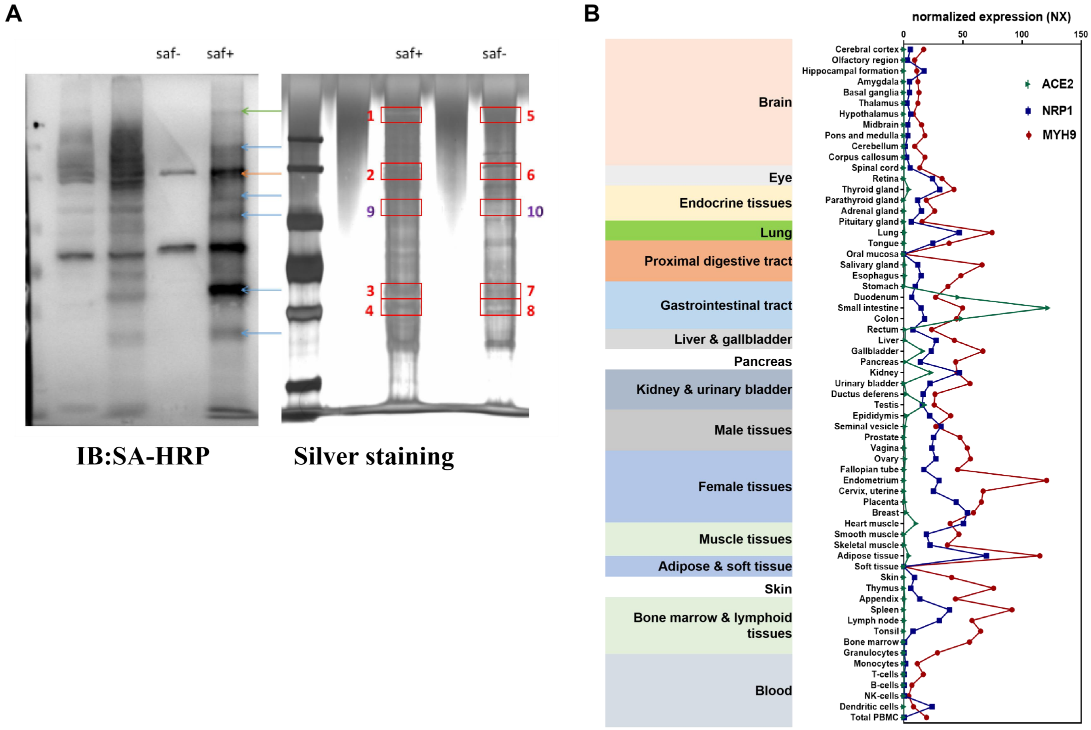

Supplementary Figure 1

MYH9 expression is high in the human lung and proximal digestive tract.

Data from Expression Atlas, Expression Atlas update - an integrated database of gene and protein expression in humans, animals and plants (Nucleic Acids Research, 2016). (A) Precipitation of the 293-ACE2 cell lysates without (line 1) or with S606-APEX2-FLAG (lane 2) treatment, analyzed by western blotting using the HRP-conjugated streptavidin and silver staining. (B) The normalized expression (NX) of MYH9 in human tissues.

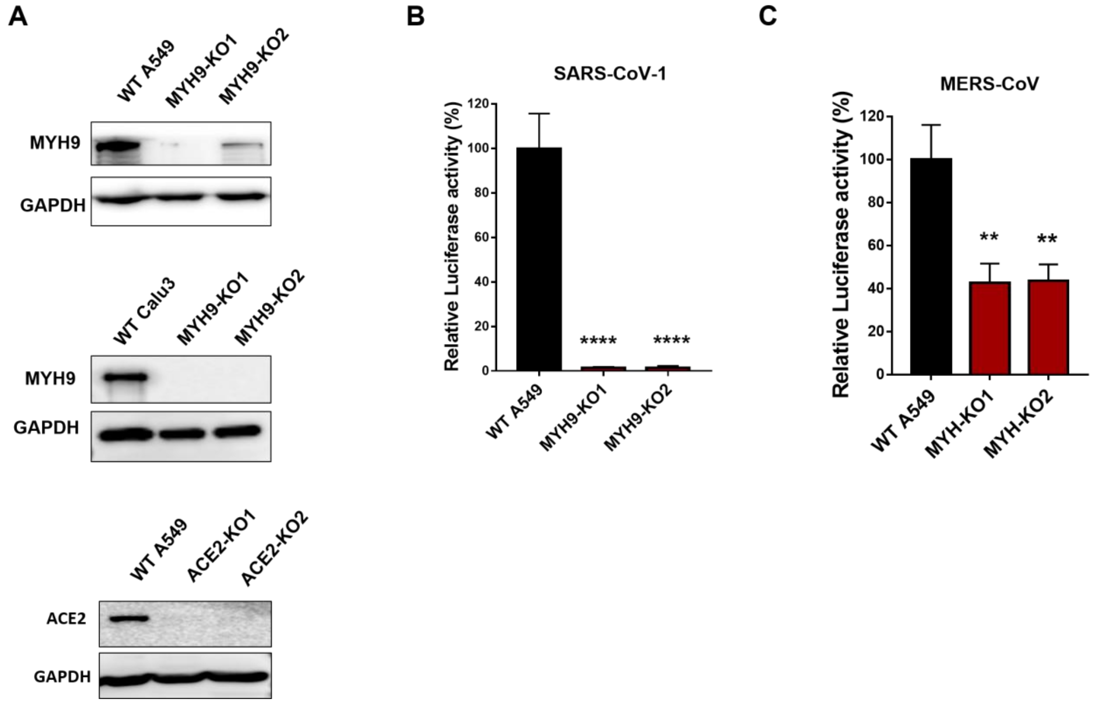

Supplementary Figure 2

MYH9 promotes the pseudovirus infection of SARS-CoV-1 and MERS-CoV in A549 cells.

(A) Immunoblot analyses of MYH9 protein levels in WT or MYH9-KO A549 (Top) and Calu-3 cells (middle). Immunoblot analyses of ACE2 protein levels in WT or ACE2-KO A549 (Bottom). The image is representative of three independent experiments. **(B-C)** Relative SARS-CoV-1(B) and MERS-CoV(C) pseudovirus infection in WT and MYH-KO A549 cells. Significant differences from WT cells were determined by Two-tailed unpaired t test. ****, $P < 0.0001$; **, $P < 0.01$. All data in this figure are presented as mean ± SEM for more than three independent experiments (n>=4).

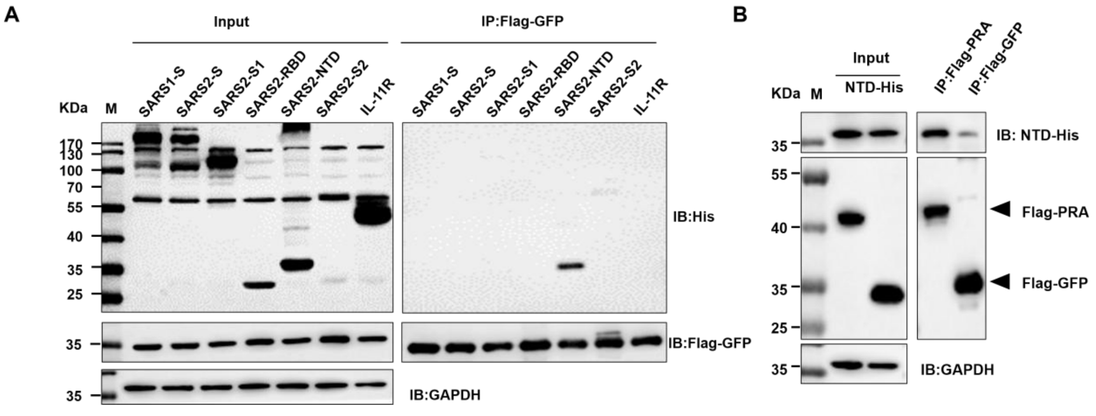

Supplementary Figure 3

（**A**）The interaction between Flag-GFP and SARS-CoV-2 spike proteins. WT 293T cells were co-transfected with Flag-GFP and His tagged SARS-CoV-1 S, SARS-CoV-2 S, S1, S2, S1-RBD, S1-NTD or IL-11R proteins as indicated, IL-11R was a negative control. Immunoprecipitation with Flag-GFP as a bait, followed by immunoblotting with anti-Flag, anti-His or anti-GAPDH antibodies. (B) The interaction between SARS-CoV-2 S1-NTD and Flag-PRA or Flag-GFP. WT 293T cells were co-transfected with His-tagged SARS-CoV-2 S1-NTD and Flag-PRA or Flag-GFP as indicated. Immunoprecipitation with Flag-PRA or Flag-GFP as a bait, followed by immunoblotting with anti-Flag, anti-His or anti-GAPDH antibodies. All experiments were conducted more than 3 times.

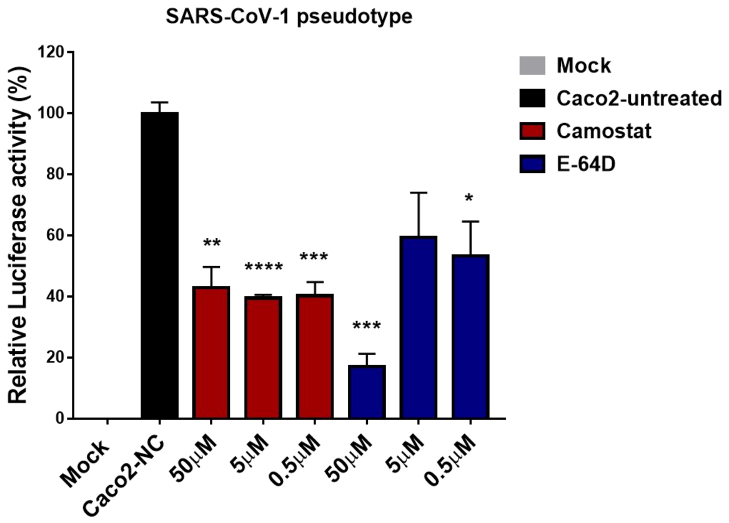

Supplementary Figure 4

Pseudovirus infection of SARS-CoV-1 in Caco-2 cells pretreated with various concentration of camostat or E-64D to inhibit serine proteases or cathepsins, respectively. Significant differences from mock treated cells were determined by Two-tailed unpaired t test; ****, $P < 0.0001$; ***, $P < 0.001$; **, $P < 0.01$; *, $P < 0.05$. All data in this figure are presented as mean ± SEM for more than three independent experiments (n>=4).

Supplementary Table1

Liquid Chromatogrphy-mass Spectrometry (LC-MS) proteins.

Accession	Description	Coverage [%]	# Peptides	# PSMs	# Unique Peptides	# AAs	MW [kDa]	calc. pI	Score Sequest HT: Sequest HT	Gene Symbol	Abundance: F1: Sample
P04264	Keratin, type II cytoskeletal 1 OS=Homo sapiens OX=9606 GN=KRT1 PE=1 SV=6	59	37	68	33	644	66	8.12	165.05	KRT1	107542215
P35579	Myosin-9 OS=Homo sapiens OX=9606 GN=MYH9 PE=1 SV=4	27	54	75	45	1960	226.4	5.6	114.6	MYH9	18473221.48
P13645	Keratin, type I cytoskeletal 10 OS=Homo sapiens OX=9606 GN=KRT10 PE=1 SV=6	37	24	43	20	584	58.8	5.21	98.71	KRT10	85526149.59
P35580	Myosin-10 OS=Homo sapiens OX=9606 GN=MYH10 PE=1 SV=3	27	51	63	42	1976	228.9	5.54	96.79	MYH10	8363841.066
P35908	Keratin, type II cytoskeletal 2 epidermal OS=Homo sapiens OX=9606 GN=KRT2 PE=1 SV=2	45	26	34	18	639	65.4	8	85.47	KRT2	19430760.1
P35527	Keratin, type I cytoskeletal 9 OS=Homo sapiens OX=9606 GN=KRT9 PE=1 SV=3	39	18	36	17	623	62	5.24	77.85	KRT9	45241571.82
P13647	Keratin, type II cytoskeletal 5 OS=Homo	25	18	22	9	59	62.3	7.74	42.25	KRT5	319251

	sapiens OX=9606 GN=KRT5 PE=1 SV=3					0					5.047
P02533	Keratin, type I cytoskeletal 14 OS=Homo sapiens OX=9606 GN=KRT14 PE=1 SV=4	21	11	20	7	472	51.5	5.16	35.08	KRT14	1939594.656
P02538	Keratin, type II cytoskeletal 6A OS=Homo sapiens OX=9606 GN=KRT6A PE=1 SV=3	16	13	17	3	564	60	8	33.82	KRT6A	537618.4375
Q14980	Nuclear mitotic apparatus protein 1 OS=Homo sapiens OX=9606 GN=NUMA1 PE=1 SV=2	6	10	10	10	2115	238.1	5.78	19.18	NUMA1	832417.5703
Q13085	Acetyl-CoA carboxylase 1 OS=Homo sapiens OX=9606 GN=ACACA PE=1 SV=2	4	8	8	8	2346	265.4	6.37	13.28	ACACA	417641.082
Q7Z406	Myosin-14 OS=Homo sapiens OX=9606 GN=MYH14 PE=1 SV=2	2	6	9	2	1995	227.7	5.6	12.14	MYH14	131917.2188
P60709	Actin, cytoplasmic 1 OS=Homo sapiens OX=9606 GN=ACTB PE=1 SV=1	17	4	5	4	375	41.7	5.48	10.82	ACTB	523236.75
Q7RTS7	Keratin, type II cytoskeletal 74 OS=Homo sapiens OX=9606 GN=KRT74 PE=1 SV=2	6	5	6	1	529	57.8	7.71	10.61	KRT74	99792.42188
O75643	U5 small nuclear ribonucleoprotein 200 kDa helicase OS=Homo sapiens OX=9606 GN=SNRNP200 PE=1 SV=2	4	7	8	7	2136	244.4	6.06	10.17	SNRNP200	348804.375
Q86YZ3	Hornerin OS=Homo sapiens OX=9606 GN=HRNR PE=1 SV=2	6	4	4	4	2850	282.2	10.04	9.16	HRNR	139374.2031

Q9H583	HEAT repeat-containing protein 1 OS=Homo sapiens OX=9606 GN=HEATR1 PE=1 SV=3	2	4	4	4	2144	242.2	6.54	8.56	HEATR1	157062.0781
P81605	Dermcidin OS=Homo sapiens OX=9606 GN=DCD PE=1 SV=2	23	3	3	3	110	11.3	6.54	7.11	DCD	2213883.531
Q05639	Elongation factor 1-alpha 2 OS=Homo sapiens OX=9606 GN=EEF1A2 PE=1 SV=1	4	2	3	2	463	50.4	9.03	5.95	EEF1A2	376656.9063
Q5T749	Keratinocyte proline-rich protein OS=Homo sapiens OX=9606 GN=KPRP PE=1 SV=1	4	2	2	2	579	64.1	8.27	4.67	KPRP	96140.75781
